# Cephalosporin use and patient outcomes following removal of penicillin–cephalosporin cross-reactivity alerts from the electronic health record

**DOI:** 10.1128/aac.01820-25

**Published:** 2026-05-15

**Authors:** Alicia Zimmer, Jessica Zagari, Paulina Sudnik, Alexandra Yamshchikov, Samia Lopa, Sarah Spitznogle, William DePasquale, Devin Donnelly, David Dobrzynski, Jessica Stern, Stephanie Shulder, Katelyn S. Quartuccio

**Affiliations:** 1 Department of Pharmacy, University of Rochester Medical Center, Highland Hospital 6923, Rochester, New York, USA; 2 Department of Pharmacy, University of Rochester Medical Center, Strong Memorial Hospital 24403, Rochester, New York, USA; 3 Department of Medicine, University of Rochester Medical Center, Strong Memorial Hospital 24403, Rochester, New York, USA; 4 Department of Medicine Biostatistical Shared Resource, University of Rochester School of Medicine and Dentistry 6923, Rochester, New York, USA; 5 Department of Allergy/Immunology and Rheumatology, University of Rochester Medical Center, Strong Memorial Hospital 6923, Rochester, New York, USA; University of Pennsylvania Perelman School of Medicine, Philadelphia, Pennsylvania, USA

**Keywords:** penicillin allergy, allergy, cross-reactivity, penicillin, cephalosporin, antibiotics, antimicrobials, anaphylaxis, electronic health record, alert

## Abstract

Studies have shown there is minimal risk of cephalosporin cross-reactivity with penicillin (PCN), though avoidance in practice still occurs. Avoidance of cephalosporins due to reported PCN allergies can result in negative clinical outcomes. Removal of cross-reactivity alerts from the electronic health record (EHR) may increase cephalosporin utilization. The objective of this study was to evaluate cephalosporin utilization in those with documented intermediate-, high-, and severe-risk allergies following an EHR cross-reactivity alert removal. This was a retrospective, observational, pre–post review of patients with a documented intermediate-, high-, or severe-risk PCN allergy who received at least one dose of a systemic antibiotic for an indication in which a cephalosporin was first line. An EHR alert removal was implemented along with system-wide guideline updates and education. The primary outcome was the rate of cephalosporin utilization. Secondary outcomes included rates of PCN utilization, antibiotic adverse effects, and a composite of treatment failure. A total of 235 patients were included (pre = 118, post = 117). Intermediate-risk allergies made up a majority of the population (74%), with surgical site infection prophylaxis being the most common antibiotic indication (63%). Cephalosporin utilization significantly increased after alert removal (27% vs 67%, *P* < 0.001). Use of cefazolin increased by 47% (*P* < 0.001), while use of alternative agents, clindamycin and vancomycin, decreased by 12.5% (*P* = 0.032) and 13.4% (*P* = 0.021), respectively. No patients had an allergic reaction to cephalosporins. Removal of a PCN–cephalosporin cross-reactivity alert from the EHR increased cephalosporin utilization without adverse allergy-related sequelae in those with intermediate- and high-risk PCN allergies.

## INTRODUCTION

Penicillin (PCN) allergies are reported in up to 10% of the population; however, less than 5% of those individuals have clinically significant IgE-mediated reactions (1). Additionally, approximately 80% of patients who experience a true IgE-mediated reaction will lose this sensitivity after 10 years, suggesting that lifelong labeling and beta-lactam avoidance are often unnecessary (1–5). The potential allergic cross-reactivity between PCN and cephalosporin antibiotics was historically attributed to their common beta-lactam ring; however, recent literature emphasizes the role of the R1 chemical side chain as a major antigenic trigger (1, 3–8).

Beta-lactams are first-line therapy for many infections, so avoidance and use of alternative regimens can contribute to adverse sequelae such as increased rates of treatment failure, antimicrobial resistance, adverse effects, prolonged hospitalizations, surgical site infections (SSIs), higher healthcare costs, and mortality (5, 7, 9–12). Use of first-line agents may be hindered by electronic health record (EHR) cross-reactivity alerts cautioning against cephalosporin use in penicillin-allergic patients. Recent literature demonstrates that alert removal increases cephalosporin use without negatively impacting patient care or development of subsequent allergic reactions (13–16). Limited data exist for patients with documented intermediate-, high-, or severe-risk allergies (e.g., anaphylaxis and non-IgE-mediated reactions) (13). The degree to which healthcare team members are alerted to cross-reactivity also varies.

To address this gap in the current literature, we conducted a retrospective, pre–post cohort study and analyzed rates of inpatient cephalosporin utilization, associated adverse events, and clinical outcomes in intermediate-, high-, and severe-risk PCN-allergic patients before and after complete PCN–cephalosporin cross-reactivity alert removal from the EHR.

## MATERIALS AND METHODS

### Study design

This study was a retrospective observational pre–post review conducted at three hospitals within an academic medical center. In July 2023, all hospitals in the medical center removed the PCN–cephalosporin cross-reactivity alert in the EHR for all healthcare team members, regardless of PCN allergy severity. Prior to removal, prescribers and pharmacists received a best practice alert to acknowledge and override allergies if appropriate. In the post group, alerts were removed for all EHR users. To observe the effect of alert removal, the study included a pre-intervention period (1 October 2021–31 March 2022), a washout period consisting of alert removal, institutional guideline and order set updates, and systemwide education, followed by a post-intervention period (1 October 2023–31 March 2024).

### Inclusion and exclusion criteria

Patients of all ages who were admitted or seen in the emergency department were identified by an EHR allergy report and were included if they had a documented PCN allergy of intermediate-, high-, or severe-risk (Table 1) at the time of study inclusion, received at least one dose of systemic antibiotics, and remained on therapy for at least 48 hours for the treatment of a confirmed or suspected infection in which cephalosporin was indicated as a first-line agent, or had received at least one dose of a systemic antibiotic, perioperatively, for an indication of SSI prophylaxis. A random number generator was used to evaluate patients identified by the EHR allergy report for study inclusion until enough patients were included to meet power. Patients could be included once per intervention period. In cases where patients had multiple admissions or ED visits during the same intervention period, the first encounter was chosen for inclusion. Patients were excluded if they had a concomitant allergy to cephalosporins, had received systemic antibiotics within 72 hours prior to the index encounter, died, or were transitioned to hospice/comfort care within 48 hours of study inclusion.

**TABLE 1:** Penicillin allergy risk stratification^*a*^

Risk stratification	Reactions
Intermediate	Anaphylaxis, angioedema, sensation of throat closing, shortness of breath/wheezing/cough, or syncope greater than 5 years from index admission
High	Anaphylaxis, angioedema, sensation of throat closing, shortness of breath/wheezing/cough, or syncope less than 5 years from index admission; or positive skin testing less than 5 years from index admission.
Severe	Mucosal ulcers, blistering, or sloughing, serum sickness, immune-mediated organ injury, drug reaction with eosinophilia and systemic symptoms, Stevens–Johnson syndrome, toxic epidermal necrolysis, and acute generalized exanthematous pustulosis

^*a*^ Risk stratification was developed in 2018 and defined according to national literature and discussions with the institution’s allergy, immunology, and rheumatology department (17).

### Data collected

Demographic information extracted from the EHR included age (years), sex, race, ethnicity, comorbidities (cardiovascular disease, chronic kidney disease, end-stage liver disease, immunocompromised status, or diabetes mellitus), primary service at the time of antibiotic initiation, ICU admission status, and pregnancy status at the time of antibiotic initiation. PCN allergy information, including specific agents and reactions, was also collected. Systemic antibiotic therapy data collected included indication for use, antibiotic(s) administered, route, and duration of therapy. Clinical outcome information obtained by chart review included recurrent infection or mortality within 30 days from the end of therapy or readmission due to index infection within 30 days of discharge, antibiotic adverse effects (antibiotic-related allergic reaction, development of *Clostridioides difficile* infection, methicillin-resistant *Staphylococcus-aureus*, vancomycin-resistant *Enterococcus faecium*, or multidrug-resistant organism within 90 days from the end of therapy), acute kidney injury (AKI; defined as an increase in serum creatinine by >0.3 mg/dL over 48 hours or a 1.5-fold increase from baseline serum creatinine over a 7-day period, occurring at any time during the antibiotic course or within 72 hours of antibiotic completion), length of hospital stay, action taken in response to a PCN allergy within 90 days from the end of therapy (allergy consultation, receipt of skin test, receipt of oral challenge, PCN allergy de-labeled, and PCN allergy relabeled), and SSI within 30 days from the end of therapy. Antimicrobial regimen switches at any point during treatment, along with rationales for regimen switches, were also collected.

### Outcomes

The primary outcome of this study was the rate of cephalosporin utilization in patients with a documented PCN allergy. The rate was determined by the number of patients who received a cephalosporin (numerator) divided by all included patients during that intervention period (denominator). Secondary outcomes included a composite of treatment failure, defined as recurrent infection or mortality within 30 days of the end of therapy, or readmission due to index infection within 30 days of discharge, the rate of PCN utilization, action taken in response to the documented PCN allergy, length of hospital stay, antibiotic adverse effects, and SSIs.

### Statistical analysis

Demographic and other clinical variables were summarized using descriptive statistics, and continuous variables were described using mean with standard deviation (for normal variables) or median with interquartile range (for non-normal variables). Categorical variables were described using frequencies and percentages. For statistical significance, categorical variables were compared between groups (pre and post) using chi-square or Fisher’s exact test (as appropriate). Continuous variables were compared across groups using Student’s *t*-test or the Mann-Whitney *U* test as appropriate. A *P* value threshold of 0.05 was selected a priori to determine statistical significance.

The sample size for the study was determined based on the baseline rate of cephalosporin use and associated effect size projections from published literature. It was estimated that a baseline rate of 14% of patients would have intermediate-, high-, or severe-risk allergy stratifications with a 15% projected effect of increased cephalosporin use associated with the change in electronic alerts and guidelines (10, 13, 18, 19). To achieve 80% power with an alpha of 0.05, it was estimated that 234 patients (117 patients in each group) would be required to adequately power the study for the primary outcome of cephalosporin utilization; secondary outcomes were evaluated descriptively. Multivariate analyses were performed using regression models that included baseline characteristics such as age, gender, and documented PCN allergy risk stratification. Statistical analysis was conducted using SAS (version 9.4; SAS software, Cary, NC).

## RESULTS

**Fig 1:**
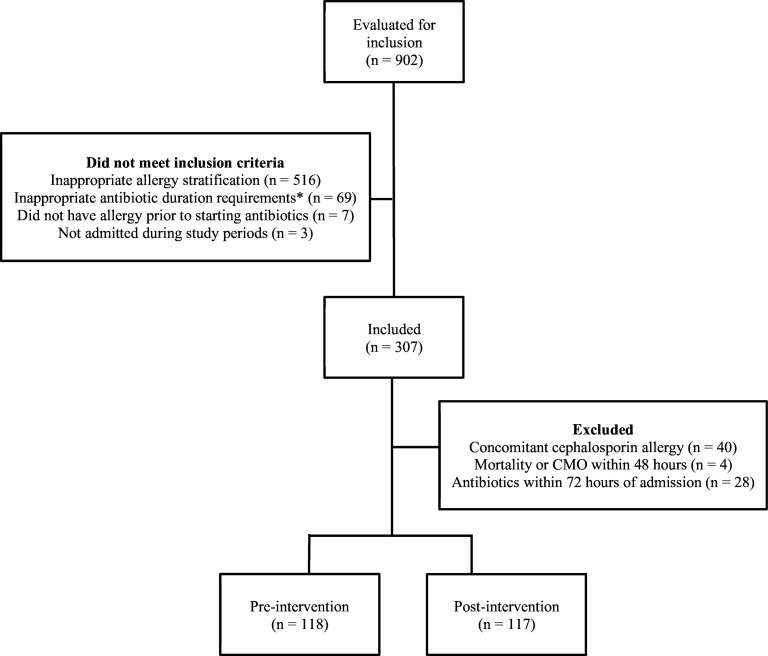
Patient screening flow diagram. *, >48 hours of systemic antibiotics for treatment of a confirmed or suspected infection in which cephalosporins are indicated or at least one dose of systemic antibiotics for surgical site infection prophylaxis. CMO, comfort measures only.

A total of 902 patients with a documented PCN allergy who received antibiotics during the observation period were evaluated for inclusion. Ultimately, 235 patients were included (pre = 118, post = 117). The presence of a concomitant cephalosporin allergy was the most common reason for exclusion (Fig. 1).

The median patient age was 60 years (IQR 43–70); 67.7% were female; 79.1% were white, and 87.7% were non-Hispanic (Table 2). Approximately half of the patients (48.5%) did not have any comorbidities, whereas 30.2% had reported cardiovascular comorbidities. There was a higher representation of intermediate-risk allergies compared to high- or severe-risk allergies in both groups (Table 2), with anaphylaxis being the most common reaction. The most common indication for antibiotic administration was SSI prophylaxis (Table 2), and the median duration of inpatient antibiotic therapy and total antibiotic duration was 2 days in both groups (IQR 1–6).

**TABLE 2:** Baseline patient characteristics^*a*^

	Pre-intervention (*n* = 118)	Post-intervention (*n* = 117)	*P* value
Age (years), median (IQR)	58 (37–72)	62 (52–70)	0.05
Sex assigned at birth			0.25
Male	34 (28.8)	42 (35.9)	
Race			0.42
White	91 (77.1)	95 (81.2)	
Black	17 (14.4)	17 (14.5)	
Other	10 (8.5)	5 (4.3)	
Ethnicity			0.84
Hispanic or Latino	7 (5.9)	6 (5.1)	
Non-Hispanic or Latino	104 (88.1)	102 (87.2)	
Unknown	7 (5.9)	9 (7.7)	
Comorbidities			
Cardiovascular disease	33 (28)	38 (32.5)	0.45
Chronic kidney disease	12 (10.2)	17 (14.5)	0.31
End-stage liver disease	2 (1.7)	2 (1.7)	>0.99
Immunocompromised	16 (13.6)	12 (10.3)	0.44
Diabetes mellitus	22 (18.6)	28 (23.9)	0.32
No comorbidities	60 (50.9)	54 (46.2)	0.47
Pregnant	8 (6.8)	2 (1.7)	0.1
Primary service			0.3
Emergency department	19 (16.1)	14 (12)	
Medicine	15 (12.7)	15 (12.8)	
Intensive care unit (ICU)	4 (3.4)	2 (1.7)	
Surgery	36 (30.5)	41 (35)	
Oncology	5 (4.2)	2 (1.7)	
Specialty	25 (21.2)	18 (15.4)	
Other	14 (11.9)	25 (21.4)	
ICU admission	10 (8.5)	12 (10.4)	0.64
Indication for antibiotics^*b*^			
Bloodstream	5 (4.2)	3 (2.6)	0.72
Bone and joint	0 (0)	2 (1.7)	0.25
Dental	3 (2.5)	2 (1.7)	>0.99
Empiric	1 (0.8)	3 (2.6)	0.37
Intra-abdominal	8 (6.8)	5 (4.3)	0.4
Respiratory	11 (9.3)	12 (10.3)	0.81
Skin and soft tissue	8 (6.8)	12 (10.3)	0.34
Surgical prophylaxis	73 (61.9)	76 (65)	0.62
Urinary	11 (9.3)	8 (6.8)	0.49
Other	10 (8.5)	6 (5.1)	0.309
Reported reaction to penicillin^*b*^			
Gastrointestinal	1 (0.8)	3 (2.6)	0.37
Anxiety	0 (0)	1 (0.9)	0.5
Rash	10 (8.5)	16 (13.7)	0.2
Urticaria/hives	22 (18.6)	25 (21.4)	0.6
Itching	7 (5.9)	1 (0.9)	0.07
Anaphylaxis	76 (64.4)	78 (66.7)	0.72
Angioedema	8 (6.8)	4 (3.4)	0.24
Sensation of throat closing	3 (2.5)	9 (7.7)	0.07
Shortness of breath/wheezing	33 (28)	37 (31.6)	0.54
Syncope	1 (0.8)	5 (4.3)	0.12
Mucosal ulcers, blistering/sloughing	1 (0.8)	1 (0.9)	>0.99
Serum sickness	1 (0.8)	0 (0)	>0.99
Immune-mediated organ injury	4 (3.4)	0 (0)	0.12
Stevens-Johnson syndrome	2 (1.7)	0 (0)	0.5
Other	12 (10.2)	19 (16.2)	0.17
Risk stratification			0.03
Intermediate	81 (68.6)	93 (79.5)	
High	32 (27.1)	24 (20.5)	
Severe	5 (4.2)	0 (0)	

^*a*^ Data reported in *n* (%), unless otherwise noted.

^*b*^ Percentages may be over 100% as patients could have had more than one antibiotic indication and more than one reported PCN reaction.

A significant increase in the rate of inpatient cephalosporin use was observed in the post-intervention period (27.1% vs 66.7%, *P* < 0.0001), specifically in the utilization of cefazolin (15.6% vs 62.8%, *P* < 0.0001) (see Table S1). The number of cephalosporin prescriptions also increased at discharge; however, this difference was not statistically significant (see Table S2). Conversely, use of clindamycin and vancomycin decreased in the post-intervention period (Table 3; Table S2). In the unadjusted analysis, patients receiving antibiotics for SSI prophylaxis during both periods had significantly lower odds of cephalosporin use (OR = 0.36, 95% CI 0.21–0.62, *P* < 0.0001) than those receiving antibiotics for other indications, and the association remained statistically significant (OR 0.255, 95% CI 0.13–0.49, *P* < 0.0001) after adjusting for race, age, comorbidity, and high-risk-allergy in a multivariate analysis (Table 4). Further investigation revealed that the increase in cephalosporin use observed post-intervention was significantly greater among those receiving antibiotics for SSI prophylaxis compared to other indications (*P* = 0.006 for the interaction between the intervention period and indication of SSI prophylaxis). More specifically, pre-OR vs post-OR (95% CI) in the subgroup receiving antibiotics for SSI prophylaxis was 0.072 (0.03–0.171) (*P* < 0.0001) compared with 0.419 (0.169–1.037) (*P* = 0.06) in the others.

**TABLE 3:** Antibiotic class received for the first course of antimicrobials

Antibiotic class received	Pre-intervention (*n* = 118)	Post-intervention (*n* = 117)	*P* value
Cephalosporin	32 (27.1)	78 (66.7)	<0.0001
Penicillin	0 (0)	1 (0.9)	0.5
Aztreonam	1 (0.8)	0 (0)	>0.99
Carbapenems	8 (6.8)	5 (4.3)	0.4
Aminoglycosides	8 (6.8)	9 (7.7)	0.79
Clindamycin	40 (33.9)	25 (21.4)	0.03
Fluoroquinolones	13 (11)	5 (4.3)	0.052
Macrolides	3 (2.5)	9 (7.7)	0.07
Metronidazole	14 (11.9)	19 (16.2)	0.34
Nitrofurantoin	0 (0)	1 (0.9)	0.5
Sulfonamides	2 (1.7)	1 (0.9)	>0.99
Tetracyclines	7 (5.9)	5 (4.3)	0.56
Vancomycin	40 (33.9)	24 (20.5)	0.02
Other	3 (2.5)	1 (0.9)	0.62

**TABLE 4:** Factors associated with cephalosporin use: multivariable analysis

Effect	Odds ratio	95% CI		*P* value
		Lower	Upper	
Pre- vs post-intervention	0.143	0.076	0.268	<0.0001
Age	1.006	0.988	1.024	0.52
Black vs other/unknown	0.66	0.161	2.701	0.83
White vs other/unknown	0.819	0.245	2.735	
Cardiovascular disease	0.846	0.416	1.719	0.64
Chronic kidney disease	2.106	0.794	5.585	0.14
High-risk PCN allergy	1.637	0.817	3.277	0.16
Receiving antibiotics for surgical prophylaxis	0.255	0.133	0.491	<0.0001

For secondary outcomes evaluating PCN utilization and response taken to PCN allergy, a single patient in the post-intervention period received a PCN antibiotic. Upon further chart review, the patient had previously tolerated PCN antibiotics, though the allergy history was never updated. One patient in the pre-intervention period underwent delabeling via a direct PCN challenge within 90 days of end of therapy with eventual discharge on oral amoxicillin. There were low rates of ADEs observed, with only a single patient in the post-intervention group experiencing a switch in antibiotics due to experiencing an AKI while on vancomycin. Overall, AKI occurred in 4 patients pre-intervention, of which 3 were receiving concomitant vancomycin, and in 10 patients post-intervention, of which 6 were receiving concomitant vancomycin.

There were no significant differences in the composite outcome of treatment failure (5.9% vs 5.1%, *P* = 0.788), or secondary outcomes, including resistant infections, SSIs, or length of hospital stay (Table 5). Notably, no patients in the study period had a documented allergic reaction associated with cephalosporin administration.

**TABLE 5:** Secondary outcomes^*a*^

	Pre-intervention (*n* = 118)	Post-intervention (*n* = 117)	*P* value
Treatment failure (composite)	7 (5.9)	6 (5.1)	0.79
Recurrent infection within 30 days of EOT	6	4	
Mortality within 30 days of EOT	1	2	
Related readmission within 30 days post- discharge	2	3	
*C. difficile* testing (within 90 days of EOT)	5 (4.2)	2 (1.7)	0.45
PCR positive	1	0	
PCR negative	4	2	
MRSA (within 90 days of EOT)	0 (0)	3 (2.6)	0.12
VRE (within 90 days of EOT)	0 (0)	1 (0.9)	0.5
MDRO (within 90 days of EOT)	3 (2.5)	2 (1.7)	>0.99
SSI (within 30 days of EOT)	5 (4.2)	1 (0.9)	0.11
Length of hospital stay (days), median (IQR)	3 (2–6)	3 (1–7)	0.52

^*a*^ Data reported in *n* (%), unless otherwise noted. Patients were included once in the composite of treatment failure outcome, though may have met more than one component criterion. EOT, end of therapy; MDRO, multidrug-resistant organism, MRSA, methicillin-resistant *Staphylococcus aureus*; PCR, polymerase chain reaction; SSI, surgical site infection, VRE, vancomycin-resistant *Enterococcus faecium*.

## DISCUSSION

This real-world interventional study demonstrated the efficacy and safety of PCN–cephalosporin cross-reactivity alert removal for improvement of guideline-driven cephalosporin utilization. While current literature primarily focuses on patients with beta-lactam allergies that are documented as lower risk, this study focused on underrepresented higher-risk allergies, such as documented angioedema or anaphylaxis, regardless of when the reaction occurred.

The significant increase in cephalosporin utilization by ~40% is similar to or higher than previously reported (13–16). This study specifically focused on indications where a cephalosporin would be considered first line, which is a unique criterion and may contribute to the larger increase from previous literature. The lack of new allergic reactions in this study is a similar finding compared to previous literature reporting no differences in adverse events or new allergic reactions in post-alert modification groups (13–16, 20). The overall low and comparable rates of antibiotic-associated adverse events observed in our study are reassuring. As this patient population consists of those with documented higher-risk allergies, this study contributes valuable information supporting the safety of cephalosporin use in PCN-allergic patients.

Previous studies have demonstrated that guideline updates and institutional education regarding the impact of side chains on cross-reactivity can potentially contribute to higher cefazolin utilization for PCN-allergic patients in the perioperative setting (21, 22). Our study is consistent with this literature as we noted a considerable increase in cefazolin use, our institution’s preferred agent for SSI prophylaxis for many procedures. Utilization of alternative agents for SSI prophylaxis has been associated with lower efficacy and a higher risk of adverse events compared to beta-lactams (5, 7, 9–12). SSIs were low among both the pre- and post-intervention groups within 30 days from the end of therapy, though this study was not powered to detect a difference in secondary outcomes. Interestingly, our analysis showed that patients receiving antibiotics for SSI prophylaxis were less likely to receive cephalosporins before and after alert removal, compared to those receiving antibiotics for other indications. Furthermore, our analysis suggested that the presence (or absence) of an alert may have been particularly impactful on antibiotic prescribing patterns in this group, with a significant interaction coefficient between the overall study effect and changes noted in prescribing rates within the SSI prophylaxis indication subgroup specifically.

Cross-reactivity alert removal not only offers the opportunity to increase utilization of first-line agents but also to reduce the incidence of adverse effects associated with alternative agents. Adverse effects in this study were evaluated based on chart review, and no severe events were reported, including cephalosporin-related allergic reactions, in either group. Cefazolin, which has minimal cross-reactivity potential compared to other cephalosporins, was the predominantly used agent in the post-intervention group, though other agents were also used without negative impact.

Given the limited number of PCN-allergic patients receiving PCNs for their first course of antibiotics and/or undergoing oral challenge in this study, we are unable to conclude whether the intervention influenced the rate of PCN utilization, or any follow-up actions to evaluate allergies, such as skin testing or delabeling efforts. The alert removal was a big step in increasing the rate of first-line agent utilization, and while there is an institutional guideline for delabeling of PCN allergies, a stronger delabeling effort could optimize antimicrobial use throughout the institution.

This study has multiple strengths. Previous literature assessing the EHR alert removal or modification often excluded or had limited representation of patients with documented high-risk allergies such as anaphylaxis, which was the most commonly reported reaction in this study (13–15). Allergies were not isolated to identification through ICD-10 codes but rather evaluated by chart review for any documented PCN allergy, including those listed as unknown or other. While this did contribute to a high degree of patient exclusion, the chart review allowed us to fully evaluate documentation to search for the allergy stratification of interest and limit allergies of lower risk and intolerances. Though severe-risk allergy stratification was not represented in the post-intervention group, there was representation of both intermediate- and high-risk stratification allergies. No patients experienced an allergic reaction, which emphasizes the safety of alert removal, even in higher-risk allergies. Additionally, a complete removal of the cross-reactivity alert from the EHR for all clinicians (including pharmacists, nurses, and ordering providers) likely further enhanced the effect of the intervention compared to alert removal for providers only.

Analysis of a retrospective pre- and post-chart review for allergic reaction documentation and explanation for antibiotic selection can be affected by confounders, though multivariate analysis may help mitigate this limitation. While thorough chart evaluation rather than identification of patients through ICD-10 codes may be a strength, reliance on chart documentation alone has the potential for inaccurate allergy descriptions and/or classifications. In addition, the retrospective design of this study did not always allow for identification of the prescribers’ rationale for utilizing alternative agents. Lack of a control or matched group may hinder demonstration of a directly causal relationship between EHR alert removal and the observed increased rate of cephalosporin utilization. Given the time elapsed between the pre-intervention and post-intervention periods, seasonality of infections and prescribing patterns of various providers and services may have contributed to the difference in observed rates of cephalosporin utilization. Lower representation of non-surgical patients in this study may limit its generalizability outside this patient population; however, demonstration of high impact in optimizing cefazolin use as first-line SSI prophylaxis can be considered a study strength. Although the rates of secondary outcomes were similar between the pre- and post-intervention in this study, larger studies are needed to evaluate significant impacts on outcomes, such as treatment failure, length of hospital stay, or recurrent infection. Though we did observe a significant decrease in vancomycin use in the post-intervention group, interestingly, AKI occurred more frequently. Alternative common AKI etiologies, such as hypovolemia, hypoperfusion, and non-antimicrobial nephrotoxic medication use, were not assessed, and we therefore are unable to draw conclusions on this finding. Lastly, patients with a severe-risk allergy, such as a Stevens–Johnson reaction or other reactions on the severe cutaneous adverse reaction spectrum, were not represented in the post-intervention period. Patients who have experienced these reactions secondary to PCN administration continue to be underrepresented in current clinical literature, and larger clinical and translational efforts are needed to assess and analyze the cross-reactivity potential of alternative beta-lactam-based regimens.

### Conclusion

Removal of the PCN–cephalosporin cross-reactivity alert from the EHR was associated with increased cephalosporin utilization without emergence of allergy-related adverse sequelae. Future considerations include addressing the current gap in clinical literature regarding cephalosporin utilization in patients with severe-risk PCN allergies.

## SUPPLEMENTAL MATERIAL

Supplemental material aac.01820-25-s0001.docx Tables S1 and S2.

ASM does not own the copyrights to Supplemental Material that may be linked to, or accessed through, an article. The authors have granted ASM a non-exclusive, world-wide license to publish the Supplemental Material files. Please contact the corresponding author directly for reuse.
