# Supplementary material for: Cephalosporin use and patient outcomes following removal of penicillin–cephalosporin cross-reactivity alerts from the electronic health record
Source: Antimicrob Agents Chemother. 2026 May 15;70(6):e01820-25. doi: 10.1128/aac.01820-25 (PMC13231902; doi:10.1128/aac.01820-25)
Supplement: Supplemental material — Tables S1 and S2. [file aac.01820-25-s0001.docx]

SUPPLEMENTAL MATERIAL

eTable 1: Cephalosporin and penicillin agent given for first course

|  | Pre-intervention  (n = 118) | Post-intervention  (n = 117) | p-value |
| --- | --- | --- | --- |
| Cephalosporin*  Cefazolin  Cefepime  Cefoxitin  Cefpodoxime  Ceftriaxone  Cephalexin | 32 (27.1)  5  8  0  2  21  2 | 78 (66.7)  49  16  1  4  18  5 | < 0.0001  < 0.001  0.08  0.50  0.45  0.6  0.28 |
| Penicillin (amoxicillin/clavulanate) | 0 (0) | 1 (0.9) | 0.50 |

Data reported in n (%), unless otherwise noted.

*Patients could have received more than one cephalosporin during the first course of treatment (switching agents to broaden or narrow therapy or for IV to PO transition).

eTable 2: Antibiotic received at discharge

|  | Pre-intervention  (n = 118) | Post-intervention  (n = 117) | p-value |
| --- | --- | --- | --- |
| Received antibiotics at discharge | 28 (23.7) | 21 (17.9) | 0.28 |
| Antibiotic received  Penicillin  Cephalosporin  Carbapenem  Clindamycin  Fluroquinolone  Macrolides  Metronidazole  Nitrofurantoin  Sulfonamides  Tetracyclines | 1  8  1  6  5  0  4  1  3  5 | 1  10  1  4  2  1  3  0  3  0 | >0.99  0.61  >0.99  0.75  0.45  0.50  >0.99  >0.99  >0.99  0.06 |

Data reported in n (%), unless otherwise noted.

*Patients could have received more than one antibiotic at discharge
